# Lunacy Laws and Asylums
‡Remarks on the Lunacy Laws, and the Asylums of England and France. By John Webster, M.D., F.R.S. London: 1856. Reprint from the *Psychological Journal*.


**Published:** 1856-10

**Authors:** 


### THE EPITOME OF SANITARY LITERATURE.

### LUNACY LAWS AND ASYLUMS.‡

DR. WEBSTER, in contrasting the Lunacy Laws and the Asylums of England and France, argues that the British

---

‡ Remarks on the Lunacy Laws, and the Asylums of England and France. By JOHN WEBSTER, M.D., F.R.S. London: 1856. Reprint from the *Psychological Journal.*

legislature might take useful lessons from their neighbours, specially with regard to the forms required for conveying lunatics rapidly to the protection of an asylum. The law of Interdiction in Scotland for those who, from "weakness, facility, or profusion," are liable to imposition, is dwelt on also by Dr. Webster, as well worthy the attention of Parliament in reference to its application to England.

